# New Jersey Echoes

**Published:** 1903-02

**Authors:** 


### NEW JERSEY ECHOES.

The New York contingent was unusually large at the meeting of the Association in New Jersey. Among those present were Drs. R. R. Bell, Berns, Ackerman, Robertson, Ellis, and V. A. Moore.

When President Lowe's term expires he should be placed at the head of the Executive Committee, where his wide experience would be most felt and most valuable to the profession in New Jersey.

Drs. Lockwood, King, and Hurley represented the old association which for many years was the active representative of the profession in the Seashore State.

Dr. H. P. Turner, of Lahaska, Pa., is a constant attendant at the meetings of the New Jersey State Veterinary Medical Association.

